# Elastic Moduli of Avian Eggshell

**DOI:** 10.3390/biology10100989

**Published:** 2021-09-30

**Authors:** Pei-Lin Chiang, Yu-Chien Tseng, Hsiao-Jou Wu, Shu-Han Tsao, Shang-Ping Wu, Wei-Cheng Wang, Hsin-I Hsieh, Jia-Yang Juang

**Affiliations:** 1 Department of Mechanical Engineering, National Taiwan University, Taipei 10617, Taiwan; pelin0817@gmail.com (P.-L.C.); r08522508@ntu.edu.tw (Y.-C.T.); r08B21004@ntu.edu.tw (H.-J.W.); heidi031158@gmail.com (S.-H.T.); r05522513@ntu.edu.tw (S.-P.W.); 2 Department of Life Science, National Taiwan University, Taipei 10617, Taiwan; 3 Taipei Zoo, Taipei 11656, Taiwan; crz81@zoo.gov.tw (W.-C.W.); dwx03@zoo.gov.tw (H.-I.H.)

**Keywords:** avian eggshell, mechanical properties, nanoindentation, texture, microstructure, electron backscatter diffraction (EBSD), elastic modulus

## Abstract

**Simple Summary:**

Birds have existed on Earth for over one hundred million years, and the eggshell is one of the main factors in them having survived for such a long period of time. The avian eggshell is a multifunctional thin-shelled structure that protects the developing embryo from damage and excessive water loss, provides the embryo with calcium for its skeleton, and sustains the weight of incubating birds. It must also be breakable for the hatchling to emerge. Elastic modulus (Young’s modulus) is the most fundamental mechanical property for such a load-bearing structure. Despite extensive studies on avian eggs, our understanding of the elastic moduli and structure–function relationship of avian eggshells remains incomplete—most previous works have focused on chicken or only a few species. One challenge is the availability and collection of freshly-laid egg samples. The present study is based on 700 freshly-laid eggs collected over a period of seven years from 2015 to 2021, covering a wide taxonomic scale and egg mass (from 1 g to 1459 g). With this large dataset, we may obtain a bird’s-eye view of the elasticity and structure–function relationships of the avian eggshell.

**Abstract:**

We analyze 700 freshly-laid eggs from 58 species (22 families and 13 orders) across three orders of magnitude in egg mass. We study the elastic moduli using three metrics: (i) effective Young’s modulus, *E*_FEM_, by a combined experimental and numerical method; (ii) elastic modulus, *E*_nano_, by nanoindentation, and (iii) theoretical Young’s modulus, *E*_theory_. We measure the mineral content by acid-base titration, and crystallographic characteristics by electron backscatter diffraction (EBSD), on representative species. We find that the mineral content ranges between 83.1% (Zebra finch) and 96.5% (ostrich) and is positively correlated with *E*_FEM_—23.28 GPa (Zebra finch) and 47.76 GPa (ostrich). The EBSD shows that eggshell is anisotropic and non-homogeneous, and different species have different degrees of crystal orientation and texture. Ostrich eggshell exhibits strong texture in the thickness direction, whereas chicken eggshell has little. Such anisotropy and inhomogeneity are consistent with the nanoindentation tests. However, the crystal characteristics do not appear to correlate with *E*_FEM_, as *E*_FEM_ represents an overall “average” elasticity of the entire shell. The experimental results are consistent with the theoretical prediction of linear elasticity. Our comprehensive investigation into the elastic moduli of avian eggshell over broad taxonomic scales provides a useful dataset for those who work on avian reproduction.

## 1. Introduction

Nature is filled with various biological materials, each having a unique design to survive in different environments. Thin-shelled structures are ubiquitous in nature, e.g., eggshells and conch shells, as they have the merit of using minimum material to construct a secure protective structure. While eggshells and conch shells are both mostly composed of calcium carbonate (CaCO_3_) with fractions of organic constituents (proteins), they have distinct microstructures in their formation. The conch shell has a crossed laminar structure whose major function is to protect the mollusk [1]. The avian eggshell is multifunctional—it allows gas and water exchange [2,3], prevents microbial contamination [4,5,6], and provides calcium for the developing embryo [7,8,9]. In addition, most birds practice bird–egg contact incubation and “egg turning” to maintain a proper condition for embryonic development [10]. Thus, eggs must sustain the weight of the incubating bird (even the massive extinct elephant birds [11]) and resist possible impact between the egg and its surroundings. Since eggshell is a load-bearing structure, its elastic modulus (or Young’s modulus) is of profound importance, as it determines how much the shell will deform under external load and is related to its strength, stability, and toughness [12]. A wealth of studies on the elastic modulus of avian eggshells have been reported in the literature [13,14], using various methods from the static compression test, the most commonly used, to vibration measurements [15]. However, the measurement of the elastic modulus of an eggshell is not straightforward, as the shell is brittle and curved. Thus the reported values often varied greatly for the same species [15]. In addition, most of those studies were concerned with a few common species only, especially chicken [16,17,18,19]. While there are over 10,000 bird species on Earth [20], with eggshells of different appearances and textures, a comprehensive comparison of elastic moduli across a broader taxonomic scale is still lacking. Additionally, our understanding of the structure–function relationships in avian eggshells remains limited.

The elastic modulus of biological materials is, in general, anisotropic and non-homogenous, and depends on chemical composition, mineral content, porosity, microstructure, and crystal structure [21,22,23,24,25,26]. Flores-Johnson et al. [24] studied the fruit of the Cocoyol palm tree; they revealed a complex hierarchical structure in the Cocoyol shell, which made the shell hard and tough. Troncoso et al. [25] studied seashells of six species, and compared their calcite- and aragonite-based layers with the mineral building blocks. They found that the different polymorphs of CaCO_3_ exhibited different microstructures, resulting in different mechanical properties. Some special microstructures are also found in lobster exoskeletons, antler bone, silica sponge, etc. [26]. In our previous study [14], we analyzed 400 freshly laid intact egg samples from 40 species (16 families and 11 orders), and discovered that the effective Young’s moduli, *E*_FEM_, of the shells were largely constant (32 ± 5 GPa). Although *E*_FEM_ is largely invariant across a wide range of egg mass, considerable variations still exist. For example, ostrich eggshell has a much larger modulus of 47.76 GPa. Can this be attributed to its mineral content, microstructure, or crystallographic characteristics?

The present study is different from our previous study [14] in three ways. First, it covers more species and egg samples (700 freshly laid eggs from 58 species belonging to 22 families and 13 orders). Second, it includes material characterizations. Third, it includes a discussion of the relationship between theoretical elastic constants (single-crystal calcite) and measured values for eggshells. We aim to obtain the elastic moduli of those eggshells and to have a better understanding of their correlations with mineral content, microstructure, and crystallographic characteristics. We use acid-base titration to measure the weight percentage of CaCO_3_ in eggshells. We measure the microstructure and crystallographic texture by scanning electron microscopy (SEM) and electron backscatter diffraction (EBSD). EBSD is particularly powerful in revealing the distribution of pores, layer structure, grain structures, and more importantly, crystal orientation [9,27]. Though EBSD has been used to study chicken [9] and maniraptoran eggshells [27], among others, there were only a few discussions that related the crystal orientation to the mechanical properties. We compare the EBSD results with those for the *E*_FEM_ and the elastic modulus, *E*_nano_, obtained from the nanoindentation test, a powerful tool that measures local properties [28]. We conduct nanoindentation along different directions, i.e., normal to the surface and on the cross-section, to obtain the anisotropic elastic moduli. We then compare the experimental results with the theoretical predictions, and discuss how mineral content, microstructure, and crystallographic texture may affect the elastic moduli of avian eggshell.

## 2. Materials and Methods

### 2.1. Egg Collection and Effective Young’s Modulus

We analyzed 700 freshly laid eggs, belonging to 58 bird species from 22 families and 13 orders (Table S1). Most egg samples were collected from Taipei Zoo and some were acquired from other sources (private farms/owners) over a period of seven years from 2015 to 2021. The method to estimate the effective Young’s modulus of eggshells was introduced in detail in our previous paper [14]. In short, we measured the egg stiffness by compression test, and calculated the *E*_FEM_ for each sample by fitting the experimental load–displacement curve using a computer simulation technique called the finite element method (FEM). In FEM, we assume a uniform thickness and homogeneous eggshell; thus, *E*_FEM_ represents the overall rigidity of the shell, including the contribution of inorganic and organic constituents as well as pores and vesicles. Note that the experimental data were corrected for machine compliance—*E*_FEM_ will be underestimated if this is not done correctly. The data of egg mass, length, width, shell thickness, and effective Young’s modulus are list in Table S1. 

### 2.2. Scanning Electron Microscope (SEM)

A Scanning Electron Microscope (SEM, Phenom G2 Pro) was used to observe the microstructure of the shells. It comprises a long-life thermionic source electron beam with an accelerating voltage of 5.0 kV, a resolution of 25 nm, and an electron-optical magnification ranging from 80 to 45,000×. The following 15 species were measured: mandarin duck (*Aix galericulata*), Egyptian goose (*Alopochen aegyptiaca*), mallard (*Anas platyrhynchos*), northern cassowary (*Casuarius unappendiculatus*), emu (*Dromaius novaehollandiae*), blue-tailed bee-eater (*Merops philippinus*), black-crowned crane (*Balearica pavonina*), gray-crowned crane (*Balearica regulorum*), red-crowned crane (*Grus japonensis*), Indian peafowl (*Pavo cristatus*), silver pheasant (*Lophura nycthemera*), zebra finch (*Taeniopygia guttata*), yellow-collared lovebird (*Agapornis personatus*), Chilean flamingo (*Phoenicopterus chilensis*), and ostrich (*Struthio camelus*).

### 2.3. Measuring Weight Percentage of CaCO_3_

The mineral content of an eggshell can be quantified by the weight percentage of CaCO_3_, which may be determined by acid-base titration. Three eggshell samples were picked for each species, and were ground into powder over a 75-μm filter. We first added 0.1 g of eggshell powder to a 25-mL 0.10-M hydrochloric acid (HCl) solution, and stirred it until the powder was completely dissolved. We then titrated the HCl solution with a 0.10-M sodium hydroxide (NaOH) solution. By recording the volume of the NaOH solution needed to reach equilibrium, the weight percentage of CaCO_3_ could be calculated. The procedure was repeated three times for each species, and the weight percentage of CaCO_3_ was obtained by averaging the three tests.

The following 16 avian species were measured: ostrich, emu, mallard, domestic chicken (*Gallus gallus domesticus*), Indian peafowl, ring-necked pheasant (*Phasianus colchicus*), domestic pigeon (*Columba livia domestica*), grey-crowned crane, Chilean flamingo, sarus crane (*Antigone antigone*), African penguin (*Spheniscus demersus*), rosy-faced lovebird (*Agapornis roseicollis*), Fischer’s lovebird (*Agapornis fischeri*), yellow-collared lovebird, blue-tailed bee-eater, and zebra finch.

### 2.4. X-ray Diffraction (XRD)

The crystal structure of the eggshell was analyzed via eggshell powder, prepared as described in the acid-base titration, with a Rigaku TTRAX 3 high-power X-ray diffractometer. The Cu Kα emission spectrum was used, which corresponded to an X-ray wavelength of 1.5406 Å, and the diffraction angle 2*θ* ranged from 20 to 60 degrees using a 0.02° step width. Comparing the XRD patterns with the database (Powder Diffraction File, JCPDS), we can determine the eggshell crystal structure. We used ostrich and chicken eggshells as representatives. We also measured the XRD pattern of synthetic single-crystal calcite (MTI Corporation) for reference.

### 2.5. Electron Backscatter Diffraction (EBSD)

In the preparation of the specimen for EBSD, the eggshell fragments were embedded in epoxy resin. Each specimen was ground with abrasive paper of 7 different grain sizes (P240, P400, P600, P800, P1200, P2500, P4000) and polished through 3 different sizes of alumina suspension (1 μm, 0.3 μm, 0.025 μm). The specimens were then coated with platinum for better conductivity. The EBSD maps were obtained using the symmetry detector attached to the field emission scanning electron microscope (JeoL JSM-7800F Prime). All EBSD data were collected (accelerating voltage: 20.0 kV; tilting of specimens: 70 degrees) and analyzed by the AZtecCrystal software.

The following 8 species were tested: Egyptian goose, mallard, northern cassowary, emu, chicken, gray junglefowl (*Gallus sonneratii*), Indian peafowl, and ostrich.

### 2.6. Nanoindentation Testing

Nanoindentation testing was performed using a Bruker Hysitron TI 950 TriboIndenter nanoindenter with a Berkovich diamond tip. The loading rate was initially 80 μN/s, which gradually increased to a maximum force of 400 μN, which was held at a constant load for 5 s, then unloaded back to 80 μN/s. The contact depth was from 60 to 100 nm. 

Similar to EBSD sample preparation, the eggshells were embedded in epoxy resin. To measure the material properties in different directions, the eggshell fragments were placed in two different orientations: one having the outer surface facing upwards (loading along the thickness direction) and the other having the cross-section facing upwards (loading normal to the thickness direction). Then they were carefully polished to make sure the surface was flat.

For loading along the thickness direction, nanoindentation was performed on multiple locations. There were 12 and 21 available indents for ostrich and chicken eggshells, respectively, after removing some unanalyzable indents due to the pop-in phenomenon that occurred during the test. For loading normal to the thickness direction, nanoindentation was performed via a 6 × 2 matrix with mutual distances of 300 μm (for ostrich) and a 5 × 3 matrix with mutual distances of 80 μm (for chicken). The distance between adjacent indents was determined by the eggshell thickness. After removing some unanalyzable indents, both chicken and ostrich samples had 12 available indents. From the load–depth curves of indentation, we could obtain the elastic constants of eggshell, denoted here as *E*_nano_. We also performed nanoindentation on the synthetic single-crystal calcite (MTI Corporation) for reference.

## 3. Results and Discussion

### 3.1. SEM Microphotograph of Avian Eggshell

Avian eggshell is a highly ordered multilayer porous material largely made of calcite crystals (a polymorph of calcium carbonate, CaCO_3_), embedded in an organic matrix, and is mainly composed of four layers from the inner to the outer surface: (i) the shell membrane, (ii) the inner mammillary cone layer adhered to the shell membrane, (iii) the columnar palisade layer, also known as the spongy layer and the squamatic ultrastructure [29], which makes up most of the shell, and (iv) a thin cuticle [30,31]. Some species, such as pigeon and budgerigar, were found to possess very thin or no cuticle [32]. The eggshells of most avian species have simple, straight pore canals that run through the shell thickness, enabling gas exchange [30]. Some species, such as ratites, possess pores branching from their origins near the shell membrane into more complex networks [33]. The palisade layer contains numerous spherical vesicles (voids) of various diameters (e.g., ~450 nm for chicken [31] and 1–2 μm for budgerigar [32]). The vesicles are not just air bubbles, but are filled with organic material connected by a continuous network of organic fibrils [34]. More SEM images can be found in ref. [35]. 

**Figure 1:**
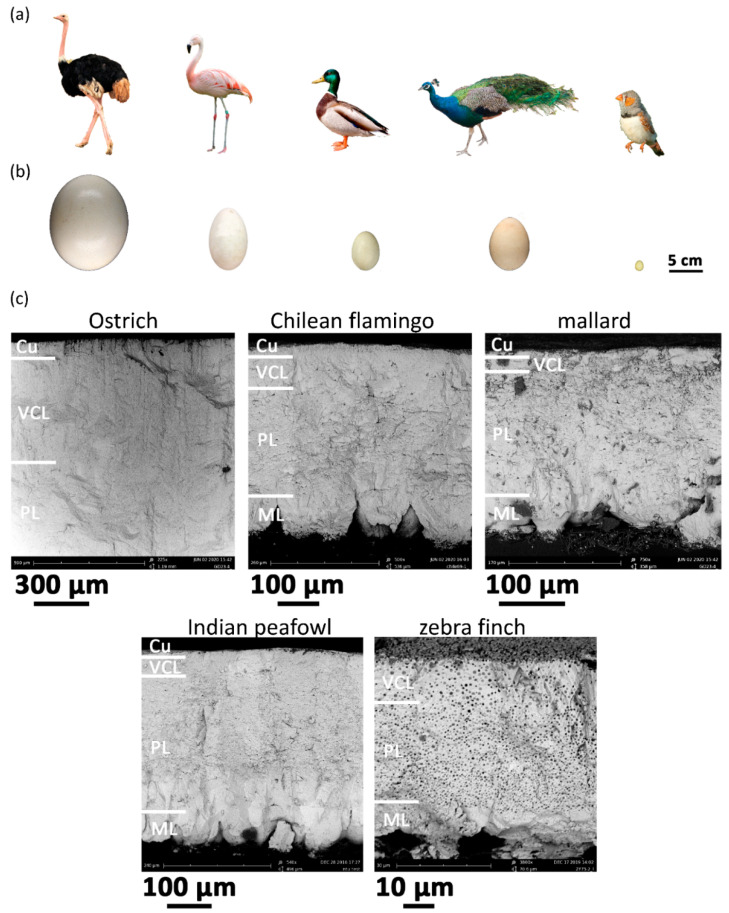
(**a**) Birds, (**b**) eggs, and (**c**) eggshell SEM images of five species. (SEM image for ostrich only contains the upper part of the shell.) Species from left to right: ostrich, Chilean flamingo, mallard, Indian peafowl, zebra finch. Abbreviations: Cu—cuticle, VCL—vertical crystal layer, PL—palisade layer, ML—mammillary layer. The copyright of avian and egg sketch images refers to Tables S2 and S3.

Figure 1 shows the birds, their eggs, and SEM microphotographs of five representative species. The eggshell of the zebra finch does not have the outer cuticle layer, and has relatively large vesicles. Because the difference in microstructure may affect the stiffness of the eggshell, we will further investigate and discuss the relationship between the microstructure and the elastic moduli of the eggshell in different ways.

### 3.2. Effective Young’s Modulus and Weight Percentage of CaCO_3_

**Figure 2:**
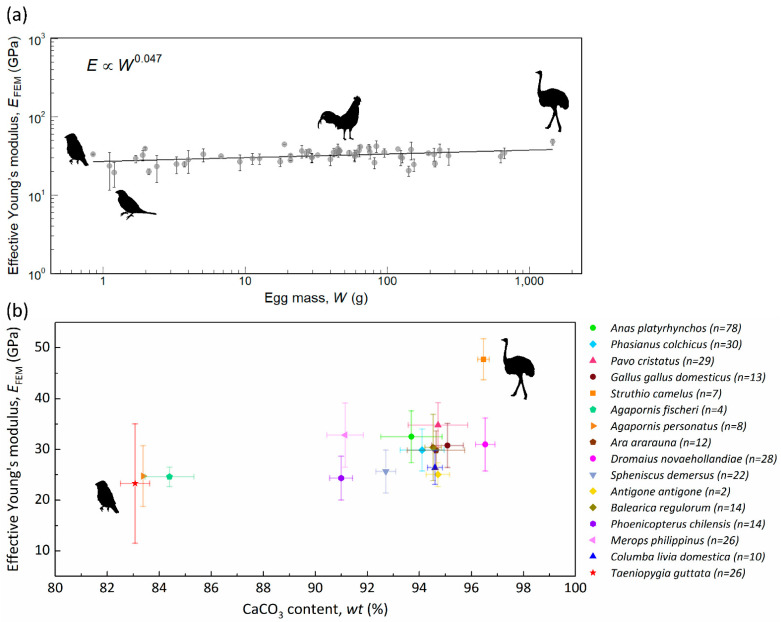
(**a**) Effective Young’s modulus, *E*_FEM_. One point represents one avian species, and the error bars are the intraspecific standard deviations. The silhouettes from left to right are zebra finch (*Taeniopygia guttata*), society finch (*Lonchura striata domestica*), domestic chicken (*Gallus gallus domesticus*), and ostrich (*Struthio camelus*). (**b**) The relation between the effective Young’s modulus and the weight percentage of CaCO_3_, and the error bars are the standard deviations. The number, *n*, is the specimen amount calculated for the effective Young’s modulus.

Figure 2a shows the effective Young’s modulus of the eggshell with respect to egg mass for 58 species. The overall average (±standard deviation) of *E*_FEM_ is 32.07 ± 5.95 GPa. The slope of the regression line is 0.047. This indicates that the effective Young’s modulus of the eggshell is largely invariant across a wide range of egg mass. Nevertheless, intraspecific and interspecific variations are still observable. The *E*_FEM_ of the society finch (*Lonchura striata domestica*) has the minimum value of 19.28 ± 6.77 GPa, and ostrich has the maximum 47.76 ± 4.03 GPa. The avian eggshell is mostly composed of calcium carbonate (CaCO_3_), with a fraction of glycoproteins and proteoglycans [36]. From previous studies, we know that the amount of minerals could affect the mechanical properties of biominerals [37]. Therefore, it is necessary to determine the weight percentage of CaCO_3_ in eggshells. We obtained the weight percentage of CaCO_3_ in eggshells of 16 species through acid-base titration (Table 1). The relation between effective Young’s modulus and weight percentage of CaCO_3_ is shown in Figure 2b. The results show that the weight percentage of CaCO_3_ ranges from 83.1% to 96.5%, and is somewhat positively correlated with *E*_FEM_. Ostrich eggshell has high CaCO_3_ (96.5 ± 0.22%) and its *E*_FEM_ (47.76 ± 4.03 GPa) is also the highest. On the other hand, the zebra finch eggshell has low CaCO_3_ (83.1 ± 0.56%) and its *E*_FEM_ (23.28 ± 11.78 GPa) is also the lowest. Note that emu eggshell also contains high CaCO_3_ (96.5 ± 0.37%), but its *E*_FEM_ (30.97 ± 5.24 GPa) is much lower than that of ostrich eggshell, which is due to the fact that emu eggshell contains a porous layer near the outer surface [38].

Bone is a biomineral with calcium phosphate being the main mineral component, and contains fractions of proteins as well as other organics such as osteopontin (OPN). Previous studies found that the Young’s modulus of osteoporotic bone, the OPN levels of which are higher, was significantly lower compared to normal bone [39,40]. Additionally, research has shown positive correlations between Young’s modulus and mineral contents [21], a result consistent with our titration tests. A lower weight percentage of calcite carbonate means a higher content of organics (lower elastic modulus) and a lower content of minerals (higher elastic modulus), resulting in a lower overall elastic modulus.

### 3.3. X-ray Diffraction

**Figure 3:**
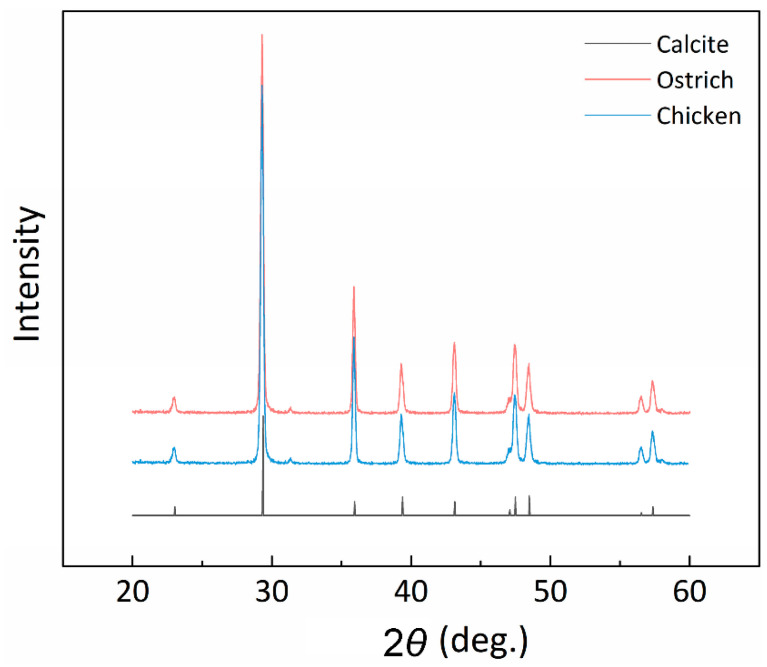
The X-ray diffraction (XRD) pattern of chicken and ostrich. The peak at the same diffracted angle of both figures reveals that the crystal of avian eggshell is calcite.

There are three polymorphs of CaCO_3_ in nature, which are calcite, aragonite and vaterite. To determine the crystal structure, we performed XRD analysis on ostrich and chicken eggshells in the form of powder. As shown in the X-ray diffraction patterns (Figure 3), the peaks of both species are at the same diffraction angle as those obtained on pure calcite, indicating that both shells are composed of calcite. This result is consistent with previous studies [41,42]. A very small peak around ~32° was observed in the samples of chicken and ostrich (but not the single-crystal calcite). This peak is likely related to the main XRD peak of the dolomite phase, as Mg has been found in avian eggshells [43].

### 3.4. Crystallographic Analysis Using EBSD

**Figure 4:**
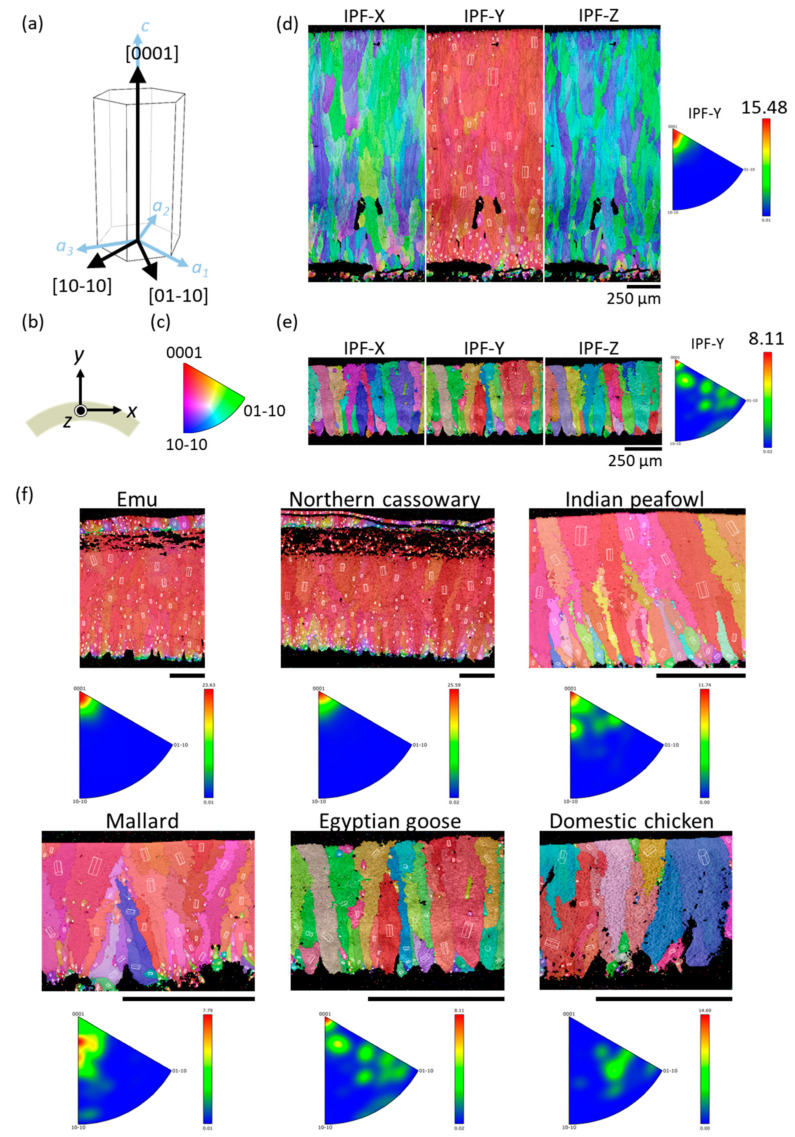
(**a**) Schematic diagram of calcite unit cell orientation with the Miller–Bravais indices. (**b**) The scanning direction of EBSD on a polished eggshell cross-section. The eggshell specimens under the Cartesian coordinate with *y*-axis pointing to the normal direction of the eggshell. The *x-* and *z*-axes are parallel to the eggshell surface. (**c**) An IPF map key shows the relationship between the color and the orientation. (**d**,**e**) The IPF maps of avian eggshell obtained by EBSD. (**d**) Ostrich; (**e**) gray junglefowl. (**f**) The IPF-*Y* maps and corresponding inverse pole figures of avian eggshell obtained by EBSD. The scale bars are 250 μm.

To find out whether crystal orientation may affect the elastic moduli of eggshells, we performed EBSD analysis on eight species, including ostrich, chicken, emu, northern cassowary, mallard, Indian peafowl, gray junglefowl, and Egyptian goose. Figure 4 displays an EBSD inverse pole figure (IPF) map of ostrich and gray junglefowl eggshells, and the rest are shown in Figure S1. Calcite crystals belong to the rhombohedral lattice system of the hexagonal crystal family, with *a* = 5 Å and *c* = 17 Å (Figure 4a). The outer cuticle is at the top of each figure, and the mammillary layer is at the bottom. The corresponding axes, indicating the directions on the eggshell, are shown in Figure 4b. The *x*- and *z*-axes are designated to be parallel to the surface, whereas the *y*-axis is in the thickness direction. The key of the IPF map indicates the crystal orientations, which can be distinguished by the triangle scheme, as shown in Figure 4c. The red endpoint refers to the [0001] direction, which is the crystallographic *c*-axis of calcite. The green and blue endpoints refer to the [01−10] and [10−10] directions, respectively.

In an IPF map, a colored area represents a grain, and colors with close resemblance indicate that the growth direction of the grains is similar. That is, if the crystals have consistent orientations, the material has a crystallographic texture, and so the colors of these crystals in the IPF map would be similar. For example, from the IPF map of the ostrich eggshell shown in Figure 4d, we observe that the colors in the IPF-*Y* map are mainly red. This means that the [0001] direction of these crystals has a strong alignment along the *y*-direction, i.e., along the thickness direction. As a result, it could be concluded that the ostrich eggshell is textured. Since the calcite in the eggshell grows from the interior to the exterior along the thickness direction [13,44,45], it can be inferred that in the growth process of the ostrich eggshell, the calcite tends to grow in the crystal [0001] direction along the eggshell’s thickness. On the other hand, the color distribution of the IPF-*X* and IPF-*Z* maps is randomly intertwined with blue and green, with no obvious patterns. This represents that the calcite crystal has no consistent orientation in the directions parallel to the surface. It could be concluded that during the formation of ostrich eggshells, the [0001] direction of calcite would orientate normally to the shell surface, but in directions parallel to the surface it is randomly distributed. 

Different species may have different degrees of crystallographic texture. For the gray junglefowl eggshell shown in Figure 4e, its IPF-*Y* map is colored with red, orange, green, and blue. The IPFs at the right of Figure 4d,e show the frequency of the crystal orientation occurrence in the *y*-direction. The color bar and the maximum value denote the frequency of the crystal orientation occurrence. Compared with the ostrich eggshell, whose crystal orientation is mostly in the [0001] direction, the crystal orientation of gray junglefowl eggshell is more spread out and is only weakly textured. However, we found that not all avian species have a textured crystal structure. For example, chicken eggshell is non-textured, as evidenced by the colorful IPF map and the IPF of chicken eggshell (Figure 4f)—there is no consistency in any direction, indicating that the calcite has no preferred orientation during the formation of chicken eggshells. This result is consistent with those obtained by Choi et al. [27] and Athanasiadou et al. [9]. An earlier study, however, reported that chicken eggshell was a crystallographic textured, and the eggs laid by younger hens were weaker than those laid by older hens [46]—this study was based on polarized light optical microscopy and X-ray diffraction (XRD), and may not have the accuracy and resolution of EBSD.

In addition, a previous study, based on optical microscopy [46], found that the mammillary layer consisted of small crystals, whereas the palisade layer consisted of bigger columnar crystals, which is similar to our results. We observed that the color distribution is more irregular towards the inner surface of the eggshell, indicating that the calcite grains generated in the primary stage of eggshell formation are smaller and more randomly oriented. As the eggshell grows, the calcite grains become bigger and grow in a more consistent direction in the palisade layer. Comparing *E*_FEM_ with the crystal orientations (texture or non-texture) of six avian species (Table 2 and Figure S2), we found that the orientation of calcite crystals appears to have no effect on *E*_FEM_. This is consistent with the result of López’s study [47], and may be explained by the fact that *E*_FEM_ measures the effective (or average) stiffness of a finite volume of eggshell that consists of many differently oriented grains. If the number of grains is large and their orientations are random enough, then variations in stiffness with individual grains may be cancelled out.

### 3.5. Nanoindentation

**Figure 5:**
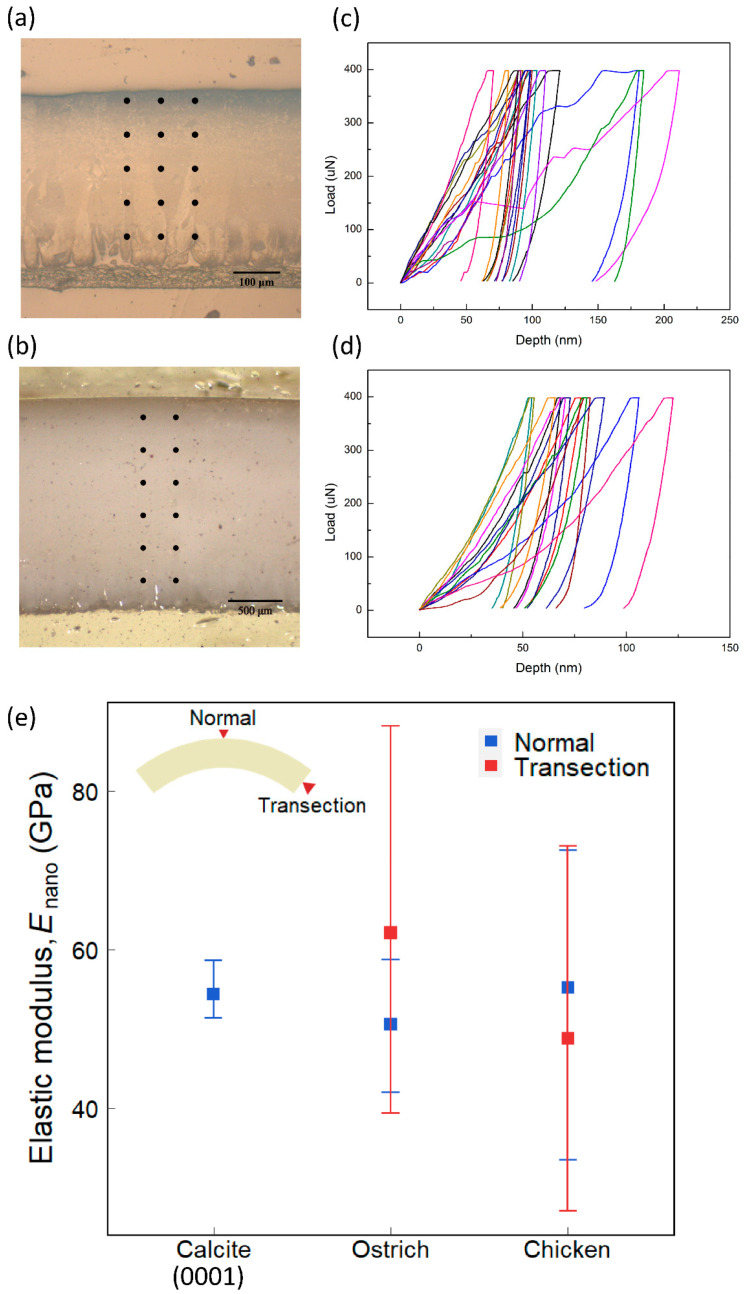
The test points in the transection direction of (**a**) chicken and (**b**) ostrich. The load–depth curves of nanoindentation on those test points for (**c**) chicken and (**d**) ostrich. (**e**) The results of nanoindentation test. The blue represents the normal direction (calcite: *n* = 4, ostrich: *n* = 12, chicken: *n* = 21). The red represents the transection direction (ostrich: *n* = 12, chicken: *n* = 11). The error bars are the intraspecific maximum and minimum values. The eggshell inset indicates the directions of the nanoindentation test.

We further carried out nanoindentation tests to measure the mechanical properties of the eggshells. From the load–depth curves of indentation (Figure 5c,d), we could obtain the elastic modulus, *E*_nano_, of eggshells [28]. A nanoindentation test was performed on ostrich and chicken eggshells in two directions—one along the thickness direction and the other perpendicular to the cross-section. Figure 5a,b shows the indentation positions in the transection direction of chicken and ostrich eggshells, respectively.

The results obtained along the thickness direction are shown as blue data points in Figure 5e. The average elastic moduli of the ostrich and chicken eggshells are 50.61 ± 5.17 GPa and 55.17 ± 9.51 GPa, respectively. Their average values are similar (~8%). The standard deviation of chicken eggshells, however, is approximately two times higher than that of the ostrich eggshell. According to the EBSD result, ostrich eggshell has a texture oriented in the thickness direction, whereas chicken eggshell does not—in this direction, the crystal orientation of chicken eggshells is more randomly arranged than that of ostrich eggshells. It is known that elastic modulus depends on the crystal orientation, which may explain the larger variation of *E*_nano_ in chicken eggshells.

The results obtained on the cross-section are shown as red data points in Figure 5e. The average elastic moduli of the ostrich and chicken eggshells are 62.12 ± 16.13 GPa and 48.75 ± 12.56 GPa. Both cases show a large standard deviation. This is due to the fact that the crystal orientations vary from grain to grain in the transection direction for both eggshells.

The effective Young’s moduli *E*_FEM_ of ostrich and chicken eggshells are 47.76 GPa and 30.77 GPa, respectively (Table 1). For both species, *E*_nano_ is larger than *E*_FEM_. Recall that *E*_FEM_ is obtained by assuming that the shell material is isotropic and homogeneous. This process averages out the position-dependent effect of anisotropy and inhomogeneity. In other words, *E*_FEM_ considers the material elasticity macroscopically, including the pores, vesicles, proteins, and other materials or defects of the eggshell. In contrast, *E*_nano_ is obtained by indenting directly on an individual calcite grain, which measures the elasticity of the calcite grain itself (in the indentation direction). Therefore, it is expected that the *E*_nano_ is, in general, larger than the *E*_FEM_ for the same specimen. In addition, out of all species, the effective elastic modulus of ostrich eggshells is significantly large. Compared with other species, ostrich eggshell is denser and has fewer defects and vesicles, resulting in a higher effective Young’s modulus, which is similar to the Young’s modulus of calcite.

### 3.6. Theoretical Values

**Figure 6:**
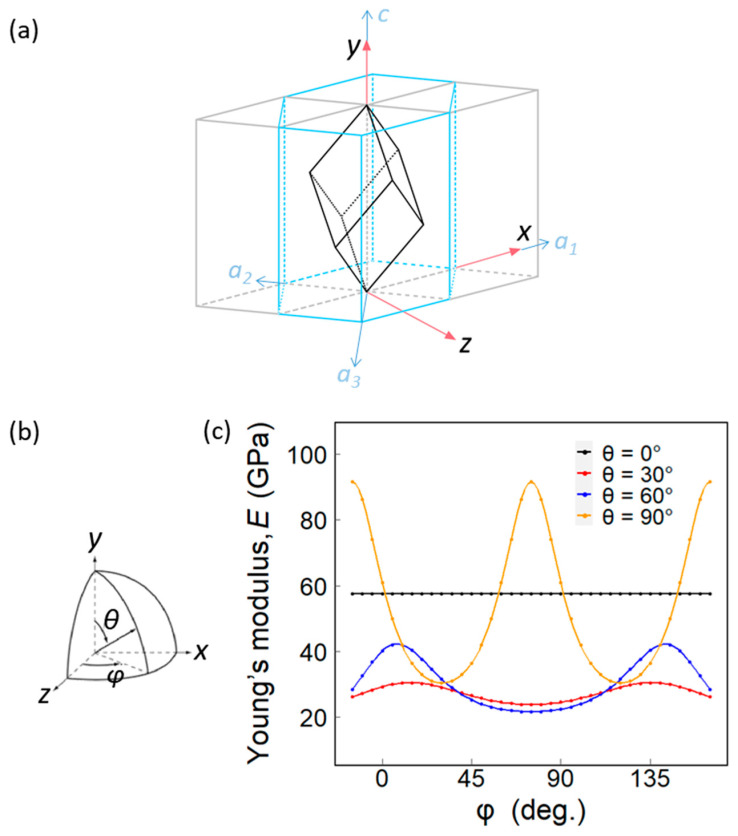
(**a**) A rhombohedral calcite unit cell is defined in the hexagonal coordinate. The parallelepiped represents a calcite lattice. The blue lines illustrate the hexagonal crystal system. (**b**) Schematic diagram of *θ* and *φ* of (**c**). (**c**) The theoretical Young’s modulus of calcite under different azimuthal angles.

The hexagonal crystal family consists of two lattice systems: rhombohedral and hexagonal. Though calcite is a rhombohedral crystal, it can be defined in the hexagonal coordinate (Figure 4a). Figure 6a shows the relationship between the two settings for the rhombohedral lattice. 

The unit cell of calcite in Cartesian coordinates has the *y*-axis pointing towards the *c*-axis of calcite. The elastic stiffness tensor of calcite [48,49] is:

(1) $$\begin{array}{l} \left[C_{ij}\right]=\left[\begin{array}{cccccc} 149.4 & 57.9 & 53.5 & 20 & 0 & 0\\ 57.9 & 149.4 & 53.5 & -20 & 0 & 0\\ 53.5 & 53.5 & 85.2 & 0 & 0 & 0\\ 20 & -20 & 0 & 34.1 & 0 & 0\\ 0 & 0 & 0 & 0 & 34.1 & 20\\ 0 & 0 & 0 & 0 & 20 & 45.75 \end{array}\right]\mathrm{GPa} \end{array}$$

The elastic compliance tensor is the inverse matrix of the elastic stiffness tensor:

(2) $$\begin{array}{l} \left[S_{ij}\right]={\left[C_{ij}\right]}^{-1}=\left[\begin{array}{cccccc} 10.92 & -3.78 & -4.48 & -8.62 & 0 & 0\\ -3.78 & 10.92 & -4.48 & 8.62 & 0 & 0\\ -4.48 & -4.48 & 17.37 & 0 & 0 & 0\\ -8.62 & 8.62 & 0 & 39.44 & 0 & 0\\ 0 & 0 & 0 & 0 & 39.44 & -17.24\\ 0 & 0 & 0 & 0 & -17.24 & 29.39 \end{array}\right]\times 10^{-3}\;\frac{1}{\mathrm{GPa}} \end{array}$$

Then we obtain the theoretical Young’s modulus, *E*_theory_, of calcite in the *x, y, z* directions:

(3) $$\begin{array}{l} E_{x}=E_{z}=\frac{1}{S_{11}}=\frac{1}{S_{22}}=91.59\;\mathrm{GPa}\\ E_{y}=\frac{1}{S_{33}}=57.59\;\mathrm{GPa} \end{array}$$

Because the *y*-axis coincides with the *c*-axis of calcite, the *E*_theory_ in the [0001] direction of calcite is 57.59 GPa. 

In addition, we may also calculate the *E*_theory_ in other crystal orientations by rotating the elastic compliance tensor in a specific direction. To do so, we put the unit cell of calcite in a spherical coordinate system, then rotate the coordinate system with the specific polar angle *θ* and azimuthal angle *φ* (Figure 6b). Through a matrix multiplication [*S*’] = [*T*_s_][*S*][*T*_s_]^T^, the *E*_theory_ in the corresponding direction can be obtained. Here, [*T*_s_] is the rotation matrix of the elastic compliance tensor, which is determined by the angles *θ* and *φ*. Figure 6c shows the *E*_theory_ calculated with a different polar angle *θ* and azimuthal angle *φ*.

Recall that the *E*_nano_ values along the thickness direction are respectively 50.61 GPa and 55.17 GPa for ostrich and chicken eggshells, which are similar to *E*_theory_ = 57.59 GPa in the [0001] direction of calcite (*θ* = 0°). According to Figure 5c, *E*_nano_ ranges from 27.01 to 88.17 GPa for the ostrich and chicken eggshells in the transection direction, which also coincides with the theoretical prediction of *E*_theory_, ranging from 21.59 to 91.59 GPa. Since the crystal orientations in the transection direction are randomly distributed, *φ* of the crystals at *θ* = 90° may be any angle, resulting in the diverse *E*_nano_ values. On the other hand, the ostrich eggshell is textured with [0001] pointing in the thickness direction, so its *E*_nano_ in the thickness direction shows relatively little variation.

## 4. Conclusions

We present a comprehensive comparative study on the elastic modulus (Young’s modulus), mineral content, and crystallographic characteristics of avian eggshells, based on 700 freshly laid eggs from 58 species, across 22 families and 13 orders. We represent the elasticity of eggshell via three different metrics: (i) effective Young’s modulus, *E*_FEM_, by compression tests and numerical simulations, (ii) elastic modulus (indentation modulus), *E*_nano_, by nanoindentation, and (iii) theoretical Young’s modulus, *E*_theory_, by the elastic compliance tensor of single-crystal calcite. Those metrics are defined differently and have different physical interpretations, but they can be compared and provide useful insights into the elasticity of eggshells. For the 58 species (700 eggs), *E*_FEM_ is 32.07 ± 5.95 GPa and is largely invariant with respect to the egg mass (across three orders of magnitude), though considerable intraspecific and interspecific variations exist. To understand the relationship between elastic moduli, mineral content, and crystallinity, we conducted acid-base titration (16 species) and EBSD (8 species) on representative species. We found that there is a slight positive correlation between CaCO_3_ content and *E*_FEM_. The ostrich eggshell has high CaCO_3_ (96.5 ± 0.22%) and its *E*_FEM_ (47.76 ± 4.03 GPa) is also the highest. On the other hand, the zebra finch eggshell has low CaCO_3_ (83.1 ± 0.56%) and its *E*_FEM_ (23.28 ± 11.78 GPa) is also the lowest. In the EBSD analysis, we found that different species exhibit different degrees of crystallographic texture—ostrich eggshell exhibits a strong texture, whereas chicken eggshell has no texture. However, the degree of texture does not appear to correlate with *E*_FEM_. We also observed that the crystal orientation is more irregular around the organic cores near the mammillary layer. In the process of eggshell formation, crystals tend to be smaller and disordered in the mammillary layer. As they grow, the crystals become larger and more aligned in the palisade layer for most species. Nanoindentation tests reveal that the *E*_nano_ values in the thickness and transection directions are different and depend on the crystal alignment (textured or non-textured), resulting from the anisotropy and inhomogeneity of the shell. The theoretical Young’s moduli of calcite in various orientations are consistent with the nanoindentation and EBSD results. Our findings provide insight into avian eggshell formation and structure–function relationships, and they may be useful for biologists working on avian reproduction and conservation, as well as for engineers working on bioinspired design and biomimetics.

## Figures and Tables

**Table 1:** Weight percentage of CaCO_3_ obtain from acid-base titration. N: number of titrations, wt.: weight percentage of CaCO3 (%), n: number of eggs, *E*_FEM_: effective Young’s modulus (GPa), S.D.: standard deviation.

Species	Common Name	N	wt. (%)	wt. S.D.	*n*	*E*_FEM_ (GPa)	*E*_FEM_ S.D.
*Agapornis fischeri*	Fischer’s lovebird	4	84.40	0.94	4	24.60	1.90
*Agapornis personatus*	Yellow-collared lovebird	1	83.38	0.00	8	24.74	5.97
*Anas platyrhynchos*	Mallard	18	93.70	1.17	78	32.50	5.11
*Antigone antigone*	Sarus crane	9	94.71	0.45	2	25.03	2.35
*Ara ararauna*	Blue and yellow macaw	3	94.64	1.10	12	29.85	3.78
*Balearica regulorum*	Gray-crowned crane	8	94.53	0.31	14	30.39	6.53
*Columba livia domestica*	Domestic pigeon	6	94.60	0.29	10	26.39	3.30
*Dromaius novaehollandiae*	Emu	6	96.53	0.37	28	30.97	5.24
*Gallus gallus domesticus*	Chicken	4	95.08	0.61	13	30.77	4.35
*Merops philippinus*	Blue-tailed bee-eater	4	91.15	0.70	26	32.81	6.30
*Pavo cristatus*	Indian peafowl	10	94.71	1.14	29	34.81	4.39
*Phasianus colchicus*	Ring-necked pheasant	15	94.11	0.84	30	29.88	4.13
*Phoenicopterus chilensis*	Chilean flamingo	9	90.99	0.44	14	24.34	4.32
*Spheniscus demersus*	African penguin	3	92.71	0.38	22	25.64	4.23
*Struthio camelus*	Ostrich	9	96.46	0.22	7	47.76	4.03
*Taeniopygia guttata*	Zebra finch	3	83.07	0.56	26	23.28	11.78

**Table 2:** Texture of six avian species from EBSD results.

Species	Common Name	Texture	*E*_FEM_ (GPa)	*E*_FEM_ S.D.	n
*Gallus gallus domesticus*	Chicken	None	30.77	4.35	13
*Alopochen aegyptiaca*	Egyptian goose	Weak	41.93	7.17	22
*Gallus sonneratii*	Gray junglefowl		36.23	1.77	3
*Anas platyrhynchos*	Mallard	Medium	32.50	5.11	78
*Pavo cristatus*	Indian peafowl		34.81	4.39	29
*Casuarius unappendiculatus*	Northern cassowary	Strong	34.60	−	2
*Dromaius novaehollandiae*	Emu		30.97	5.24	28
*Struthio camelus*	Ostrich		47.76	4.03	7

## Data Availability

The data presented in this study are available on request from the corresponding author.

## Supplementary Materials

The following are available online at https://www.mdpi.com/article/10.3390/biology10100989/s1, Figure S1: the IPF maps of avian eggshell obtained by EBSD, Figure S2: the effective Young’s modulus compared with the texture, Table S1: dataset of eggs, Table S2: copyright information about bird images, Table S3: copyright information about egg images.
