# Supplementary material for: Elastic Moduli of Avian Eggshell
Source: Biology (Basel). 2021 Sep 30;10(10):989. doi: 10.3390/biology10100989 (PMC8533214; doi:10.3390/biology10100989)
Supplement: Supplementary file 1 [file biology-10-00989-s001.zip › Supplementary.pdf]

Supplementary Material for
Elastic moduli of avian eggshell

Pei-Lin Chiang [1,†], Yu-Chien Tseng [1,†], Hsio-Jou Wu [1,2], Shu-Han Tsao [1], Shang-Ping Wu [1], Wei-Cheng Wang [3], Hsin-I Hsieh [3], and Jia-Yang Juang [1,*]

[1]Department of Mechanical Engineering, National Taiwan University, Taipei 10617, Taiwan

[2]Department of Life Science, National Taiwan University, Taipei 10617, Taiwan;

[3]Taipei Zoo, Taipei 11656, Taiwan

* Correspondence: jiayang@ntu.edu.tw (J.-Y. J.)

† These authors contributed equally to this work.

National Taiwan University

Taipei 10617, Taiwan

*Corresponding author: jiayang@ntu.edu.tw

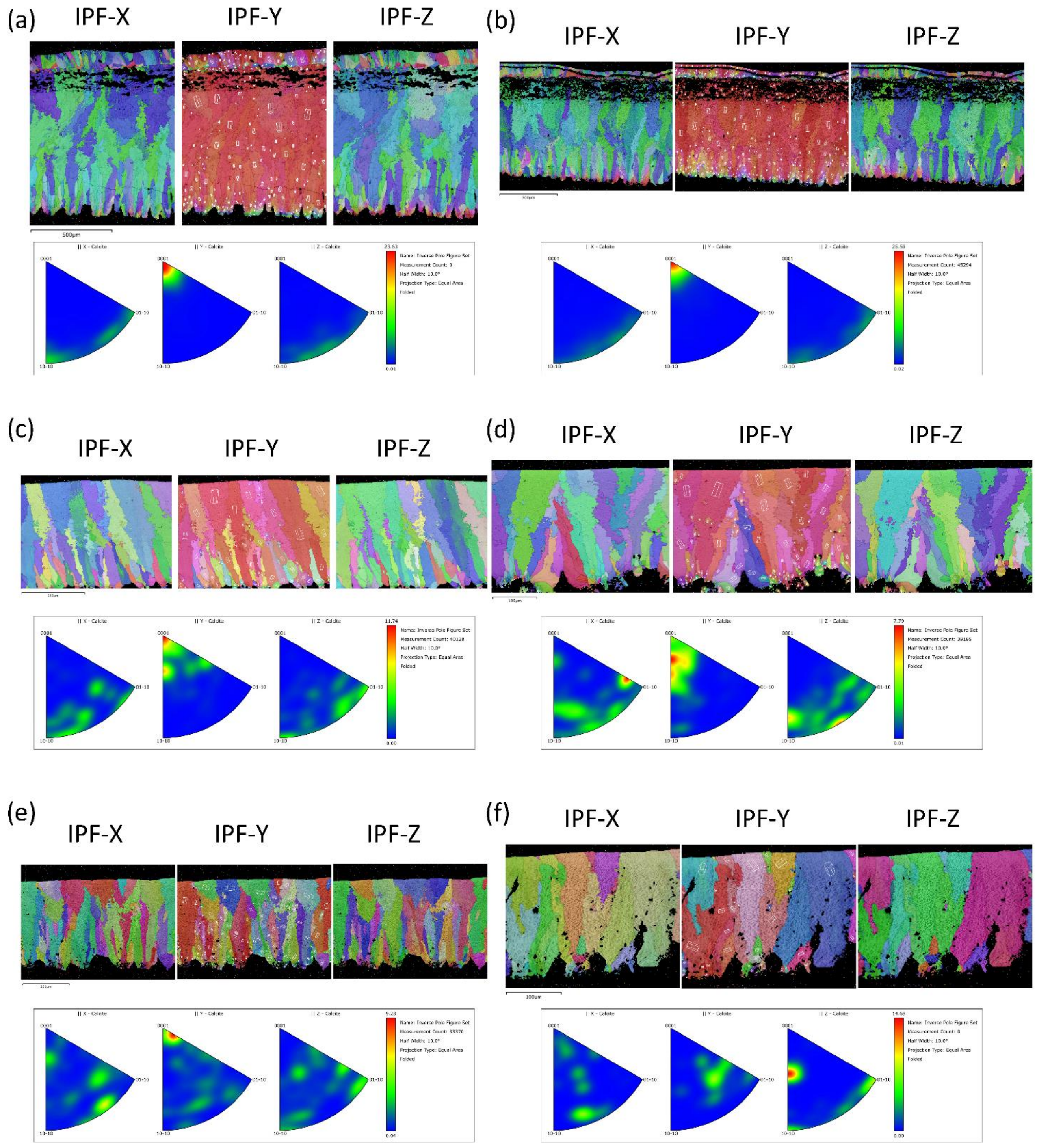

Figure S1. The IPF maps of avian eggshells obtained by EBSD. (a) Emu, (b) Cassowary, (c) Blue peacock, (d) Mallard, (e) Egyptian goose, and (f) Gray junglefowl.

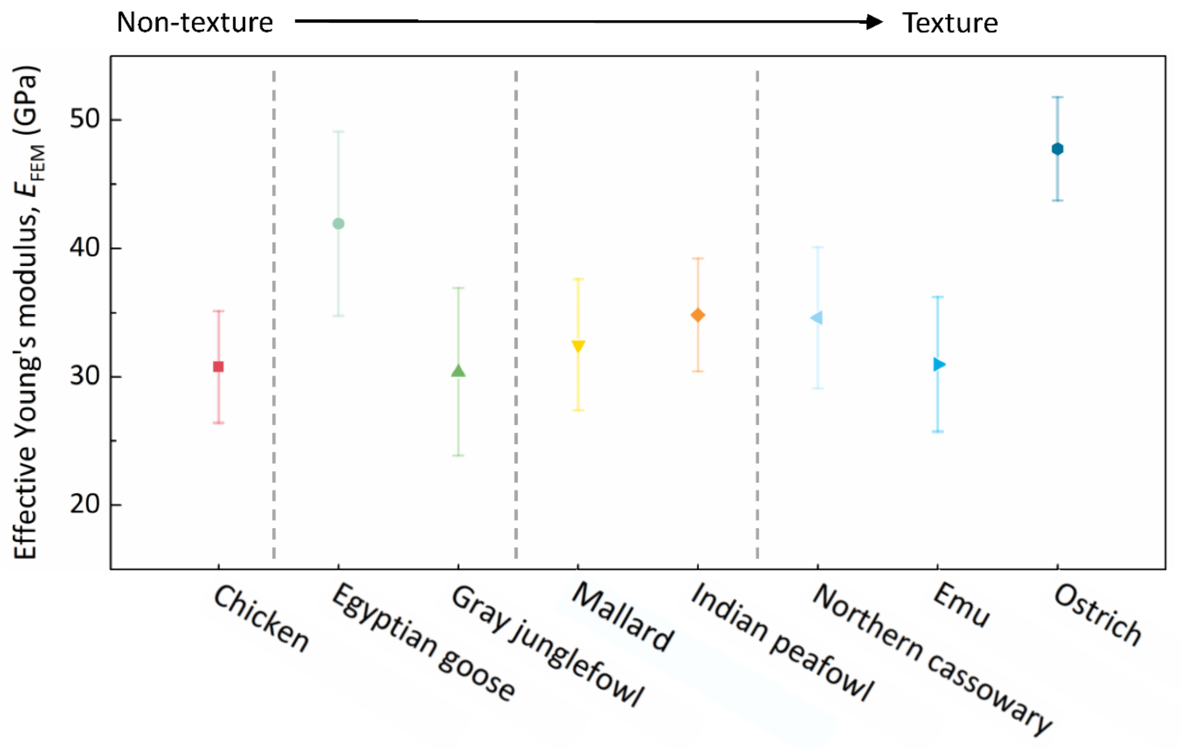

Figure S2. The effective Young's moduli versus crystallographic texture.
